# Is there a role for cardiovascular magnetic resonance imaging in the assessment of biological aortic valves?

**DOI:** 10.3389/fcvm.2023.1250576

**Published:** 2023-12-06

**Authors:** Emmanuelle Vermes, Laura Iacuzio, Sylvestre Maréchaux, Franck Levy, Claudia Loardi, Christophe Tribouilloy

**Affiliations:** ^1^ Department of Cardiology, Amiens University Hospital, Amiens, France; ^2^ Department of Cardiology, Centre Cardio-Thoracique de Monaco, Monaco City, Monaco; ^3^ GCS-Groupement des Hôpitaux de l’Institut Catholique de Lille/Lille Catholic Hospitals, Heart Valve Center, Cardiology Department, ETHICS EA 7446, Lille Catholic University, Lille, France; ^4^ Department of Thoracic Surgery, Tours University Hospital, Tours, France; ^5^ UR UPJV 7517, Jules Verne University of Picardie, Amiens, France

**Keywords:** surgical bioprosthetic aortic valve replacement, trans aortic valve implantation, cardiovascular magnetic resonance, paravalvular aortic regurgitation, myocardial fibrosis, extracellular volume

## Abstract

Patients with biological aortic valves (following either surgical aortic valve replacement [SAVR] or trans catheter aortic valve implantation [TAVI]) require lifelong follow-up with an imaging modality to assess prosthetic valve function and dysfunction. Echocardiography is currently the first-line imaging modality to assess biological aortic valves. In this review, we discuss the potential role of cardiac magnetic resonance imaging (CMR) as an additional imaging modality in situations of inconclusive or equivocal echocardiography. Planimetry of the prosthetic orifice can theoretically be measured, as well as the effective orifice area, with potential limitations, such as CMR valve-related artefacts and calcifications in degenerated prostheses. The true benefit of CMR is its ability to accurately quantify aortic regurgitation (paravalvular and intra-valvular) with a direct and reproducible method independent of regurgitant jet morphology to accurately assess reverse remodelling and non-invasively detect focal and interstitial diffuse myocardial fibrosis. Following SAVR or TAVI for aortic stenosis, interstitial diffuse fibrosis can regress, accompanied by structural and functional improvement that CMR can accurately assess.

## Introduction

The number of patients with severe aortic valve disease requiring either surgical bioprosthetic aortic valve replacement (SAVR) or transcatheter aortic valve implantation (TAVI) is increasing worldwide with the increasing aging population. For many years, SAVR has been the method of choice and a routine procedure for symptomatic patients with severe aortic valve disease or selected asymptomatic patients (1). TAVI is increasingly being used for elderly patients with severe aortic stenosis (AS) in situations of intermediate to high surgical risk (2, 3) and more recently for patients with low surgical risk (4, 5). Following either SAVR or TAVI, all patients need lifelong follow-up by imaging monitoring to detect early deterioration in prosthetic function (structural failure caused by tissue degeneration, thromboembolic complications, pannus, or infection) and ventricular function.

Two dimensional echocardiography is currently the first-line modality due to its ability to assess prosthetic heart valve function (leaflet morphology and mobility, which are Doppler –based parameters) to measure the trans-prosthetic pressure gradient and flow (6) and should be performed within 30 days after valve implantation and then yearly, according to recommendations (7, 8). In situations of a poor acoustic window or suspected prosthetic dysfunction (valve stenosis or regurgitation), transoesophageal echocardiography is considered, as well as cardiac computed tomography (CCT), to scan for additional information on pannus or thrombus (6).

Cardiac magnetic resonance imaging (CMR) is the gold-standard imaging modality for assessing ventricular volume, mass, and function, as well as an accurate and reliable tool for quantifying native valvular regurgitation (9) and, to a lesser degree, aortic stenosis (10–12). CMR is also a unique non-invasive tool for assessing focal [with late gadolinium (LGE) sequences] and diffuse fibrosis (with T1 mapping).

In the presence of biological aortic valves, CMR has been demonstrated to be safe for commonly used field strengths of 1.5 and 3 Tesla (13, 14). However, the presence of metal distorts the magnetic field, resulting in a localized signal void on cine and phase-contrast sequences. Data on CMR for patients after either SAVR or TAVI is scarce, partially due to CMR valve-related artefacts. Therefore, its utility in the assessment of prosthetic aortic valve has not been well established.

Here, we aim to provide an overview of the role of CMR in the assessment of prosthetic aortic valves (surgical and TAVI). We will detail how CMR can assess prosthetic valve function and dysfunction and evaluate the impact of the procedure on myocardial structure.

### General considerations

**Figure 1:**
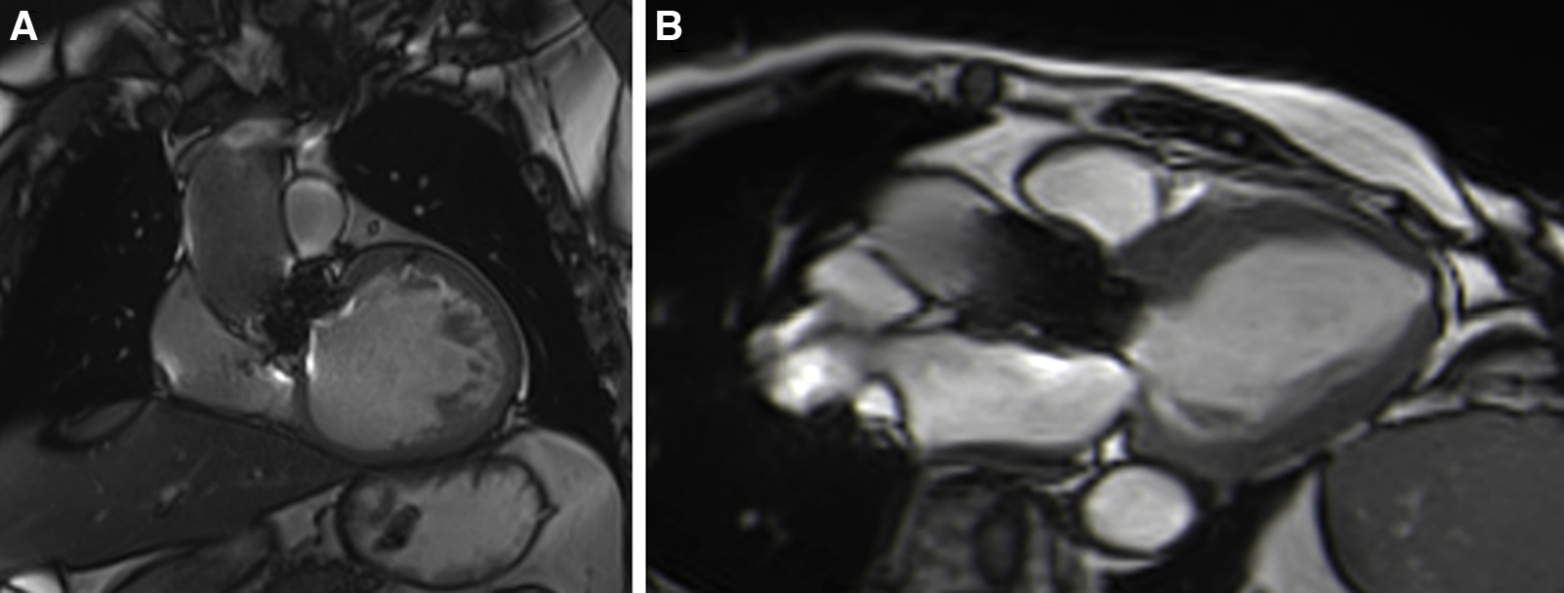
Example of a surgical stented bioprosthetic aortic valve (perimount) visualized in the coronal left ventricular outflow tract (LVOT) (**A**) and the medtronic corevalve prosthesis visualized in a three-chamber view (**B**). The ferromagnetic component of the prostheses generated important artefacts, particularly for the corevalve prosthesis.

**Figure 2:**
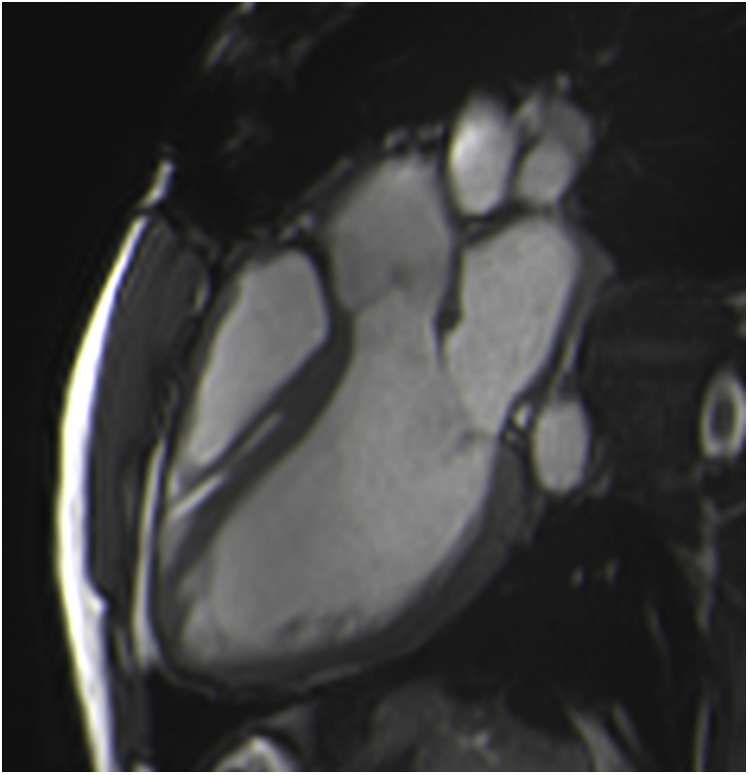
Example of a stentless aortic prosthesis (freestyle) visualized in three-chamber view bSSFP sequences, free from any artefacts and appearing as a native aortic valve.

Patients with either SAVR or TAVI can safely have CMR at 1.5 or 3 T (15). However, magnetic field inhomogeneities related to the metal component of the prosthesis generates artefacts (localized signal voids), especially with stented bioprosthetic aortic valves [made from porcine aortic valves or from bovine pericardial tissue mounted on a flexible plastic or titanium model frame (stent)] and TAVI (metallic frame with a valve mounted in the centre) (Figure 1). These artefacts often limit accurate assessment of valve planimetry and Valsalva measurements, making it impossible to identify the origin of aortic regurgitation. In contrast, stentless bioprosthetic aortic valves are made from porcine aortic roots or cryopreserved human cadaveric aortic roots with no metallic components. Thus, their appearance is consequently very similar to that of native aortic valves, without any artefacts (Figure 2), as well as that of homografts and autografts (human tissue valves). However, all the CMR acquisitions described in Table 1 remain highly challenging in clinical practice for arrhythmic patients and those who have difficulty holding their breath. These two conditions can cause motion artefacts which can blur or distort images.

**Table 1:** CMR parameters used for biological aortic valves assessment: advantages and limitations.

CMR acquisition	Usefulness/advantages	Limitations/pitfalls
Cine imaging 3-chamber Coronal LVOT Short axis covering the prosthetic valve	– visualization of aortic regurgitation – visualization of peripheral metal ring of aortic bioprosthesis – Planimetry of normal prosthetic orifice – gold standard modality for, LV mass, wall thickness ventricular volumes (without geometric assumptions) and ejection fraction – highly reproducible and accurate – accurate to assess LV reverse remodeling	– lower spatial resolution than echocardiography (less accurate to assess calcifications in degenerated aortic prosthesis) – less sensitive than echocardiography to visualize the origin of aortic regurgitation – time consuming – less accurate in case of arrhythmic disorders – limited in patients with breath holding difficulties and claustrophobia
2D phase contrast velocity imaging	– measurement of peak transprosthetic velocity and gradient – measurement of effective orifice area – quantification of paravalvular aortic regurgitation (AR vol = area under the backward flow curve in diastole; RF = ARvol/area under the forward flow curve) – independent of jet morphology – simple equation to calculate regurgitant volume and RF	– lower temporal resolution than echocardiography: underestimation of peak jet velocity and transprosthetic gradient – expertise for selecting the appropriate plane
LGE imaging Short axis LV stack 2-chamber 3-chamber 4- chamber	– unique non invasive modality to visualize and quantify focal fibrosis in the myocardium associated with cardiovascular mortality	– requires administration of intravenous contrast agent
T1 mapping Pre and post contrast (15 to 20′) Short axis view	– allows measurement of ECV: marker of diffuse interstitial fibrosis and ICV (1-ECV)	– requires recent hematocrit value and administration of intravenous contrast agent

CMR, cardiac magnetic resonance; LVOT, left ventricular outflow tract; LV, left ventricle; LGE, late gadolinium enhancement; AFF, aortic forward flow; LVSV, left ventricular stroke volume; AR vol, aortic regurgitant volume; RF, regurgitant fraction; ECV, extra cellular volume fraction; ICV, intracellular volume fraction.

### Cine imaging for biological aortic valve planimetry and thoracic aortas

Standard long-axis cine images in a three-chamber view and coronal left ventricular outflow tracts (LVOTs) are the preferred views to visualize the peripheral metal ring (Table 1). The prosthetic orifice area, independent of the flow, can be measured from a stack of contiguous cine through-plane images perpendicular to the prosthesis using two orthogonal planes (standard and coronal LVOT) covering the entire prosthesis and allowing manual delineation of the systolic orifice area.

**Figure 3:**
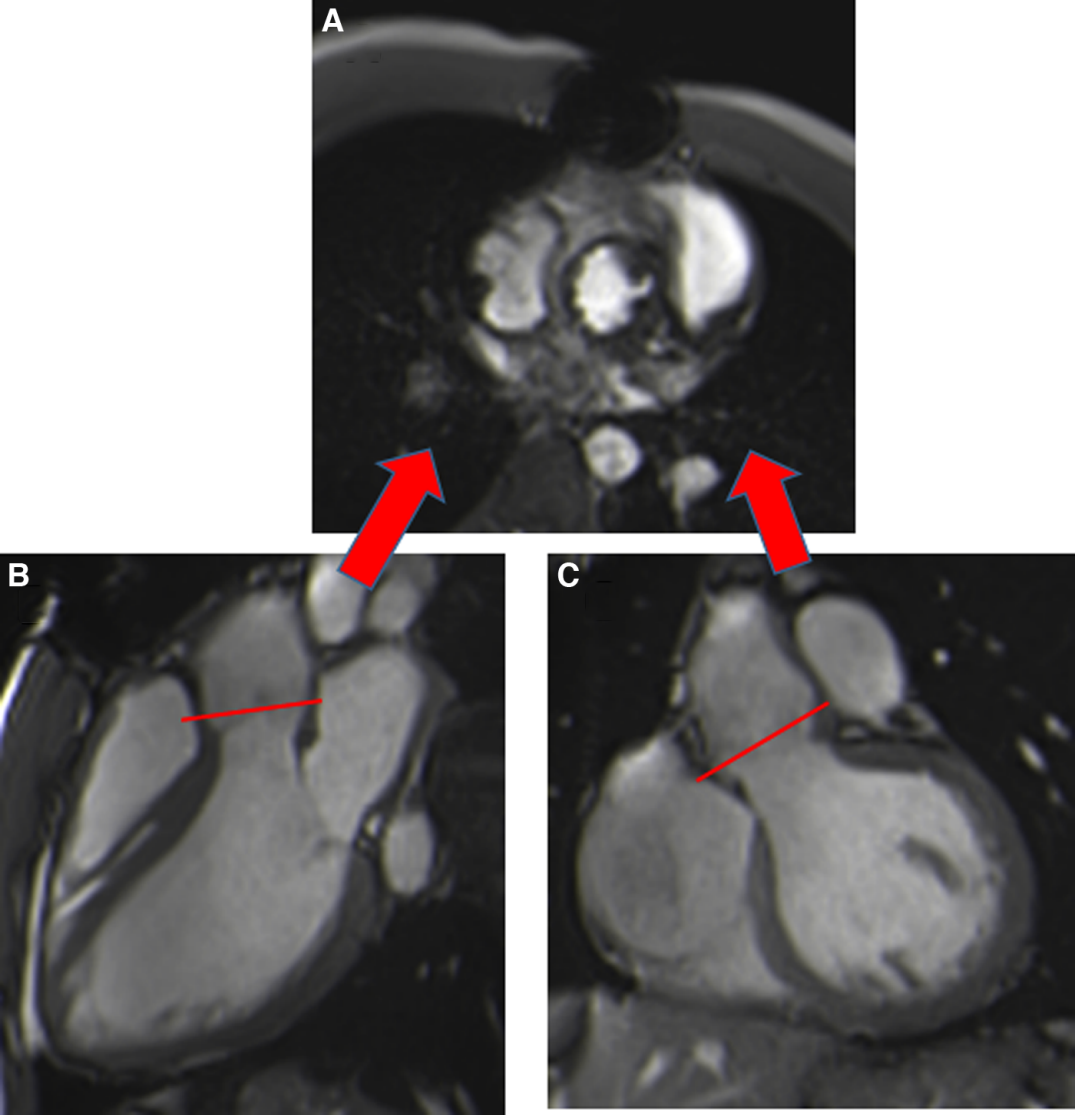
Visualization of the prosthetic orifice of a stentless aortic valve (freestyle) (**A**) obtained from two orthogonal planes: three-chamber view (**B**) and coronal LVOT (**C**). The red line indicates the slice position. Visualization of the leaflets allows easy planimetry.

**Figure 4:**
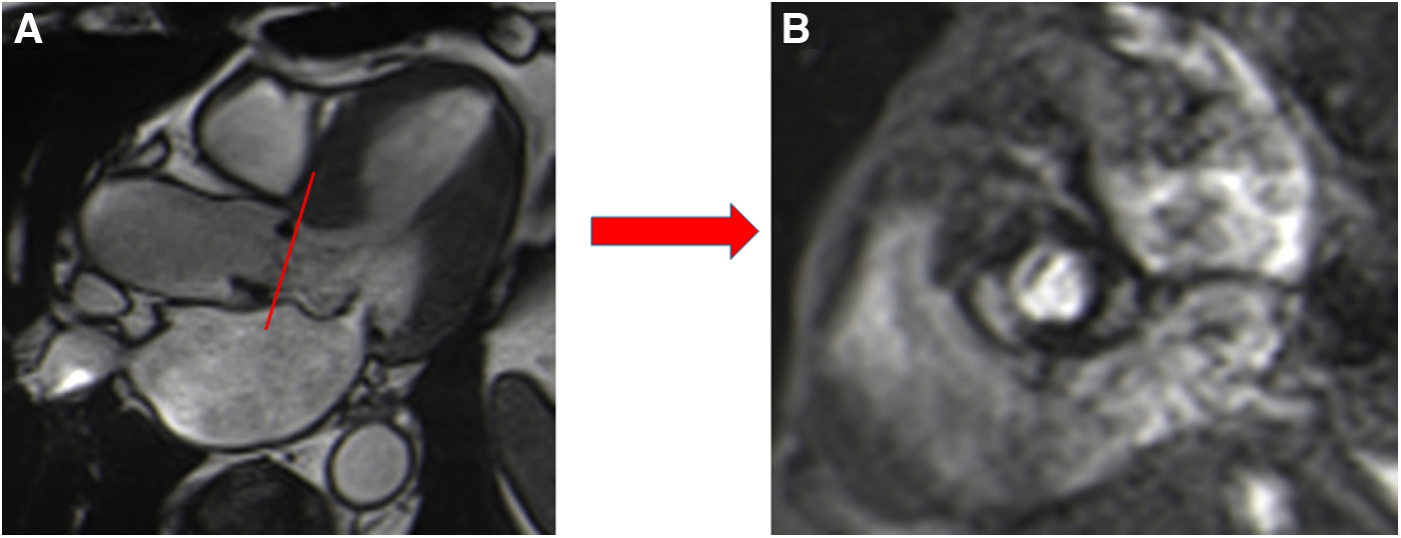
Visualization of the prosthetic orifice of a surgical stented bioprosthetic aortic valve (saint-jude 25) obtained from a three-chamber view (**A**). The visualization of leaflets for planimetry is more challenging (**B**).

Cardiac balanced steady-state free precession (bSSFP) sequences, with their high signal-to-noise ratio and precise discrimination between blood and tissue, allow biological valve delineation and orifice area planimetry for stentless bioprosthetic valves (Figure 3). For surgical stented prostheses, orifice area planimetry is more difficult due to CMR stent-related artefacts (Figure 4). A spoiled echo sequence, less prone to artefacts from ferromagnetic metals, may generate better image quality. Border discrimination of the valve leaflet is almost impossible for degenerated prostheses due to leaflet calcifications, causing additional signal void.

In the absence of specific CMR reference values, those obtained by echocardiography are used (16). A close agreement between CMR and transthoracic and transoesophageal echocardiography has been shown for surgical stented and stentless prostheses with a normal orifice area, with feasibility for 95.4% of patients but reduced image quality due to artifacts for 15.4% (17).

**Figure 5:**
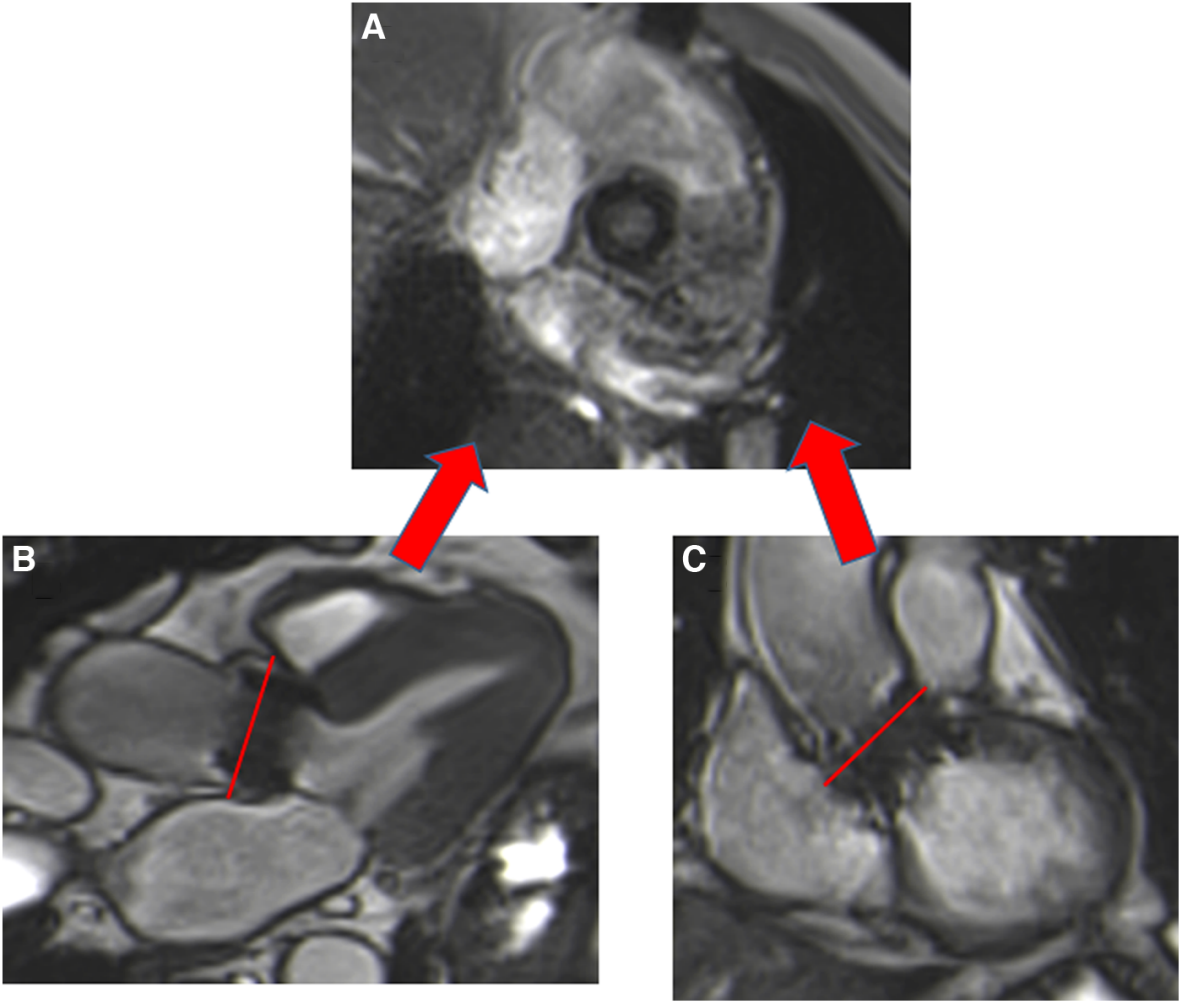
Visualization of the orifice of the Edwards-Sapien prosthesis (**A**) obtained from two orthogonal planes: three-chamber view (**B**) and coronal LVOT (**C**). CMR stent-related artefacts prevent accurate aortic valve planimetry.

Susceptibility to artefacts can be extensive for patients with TAVI, despite the absence of sternal wire artefacts, especially for the Corevalve® prosthesis due to a large stent from the LVOT beyond the sinotubular junction, making measurement of the orifice area unfeasible. Aortic orifice planimetry can also be challenging for the Edwards-Sapien® prosthesis (Figure 5).

The aorta (from the aortic roots to the descending aorta) can be visualized and measured using non-contrast-enhanced MR angiography with a respiratory navigator or breath-hold contrast-enhanced MR angiography. These measurements are particularly useful for monitoring the progression of aortic dilatation for patients with surgical treatment of bicuspid aortic valve disease or those with a biological Bentall conduit.

### Phase contrast CMR for transvalvular blood-flow velocity and effective orifice area measurements

Peak trans-prosthetic velocity and transvalvular gradients can be measured using phase contrast (PC) sequences in a through plane acquired in the ascending aorta (above artefacts generated by the aortic prosthesis) with appropriate velocity encoding (to avoid aliasing), generating phase and contrast images. Delineation of the aorta generates a peak velocity curve that allows calculation of the forward trans-prosthetic jet velocity (Table 1). However, the peak jet velocity is generally underestimated by CMR due to its lower temporal resolution relative to echocardiography and a dependence on plane selection (10, 11), especially with CMR 4D flow (18). By contrast, the effective orifice area (EOA), used as a measure for the assessment of stenosis and an important parameter to diagnose prothesis-patient mismatch, may be more robust (19). Maragiannis et al. compared the EOA by echocardiography in a cohort of 38 patients with surgical aortic valve replacement to the PC- EOA by CMR, calculated as the forward flow volume divided by the PC-based velocity time interval (VTI) (20). The forward flow volume was obtained from the PC sequence at the ascending aorta or sinotubular junction and the constructed VTI curves were based on 11 data points during the systolic ejection period at the vena contracta area. Although the PC-derived VTI was slightly smaller (bias −1.18 cm) than the Doppler-derived VTI, the authors found good agreement in the classification of aortic valve stenosis severity (*ǩ* = 0.826). This CMR method could be considered for assessing aortic valve function of surgical bioprostheses when echocardiography methods are inconclusive or discordant with the clinical presentation. Assessment of the EOA by CMR in TAVI has not yet been investigated.

### Phase-contrast CMR for paravalvular aortic regurgitation

**Figure 6:**
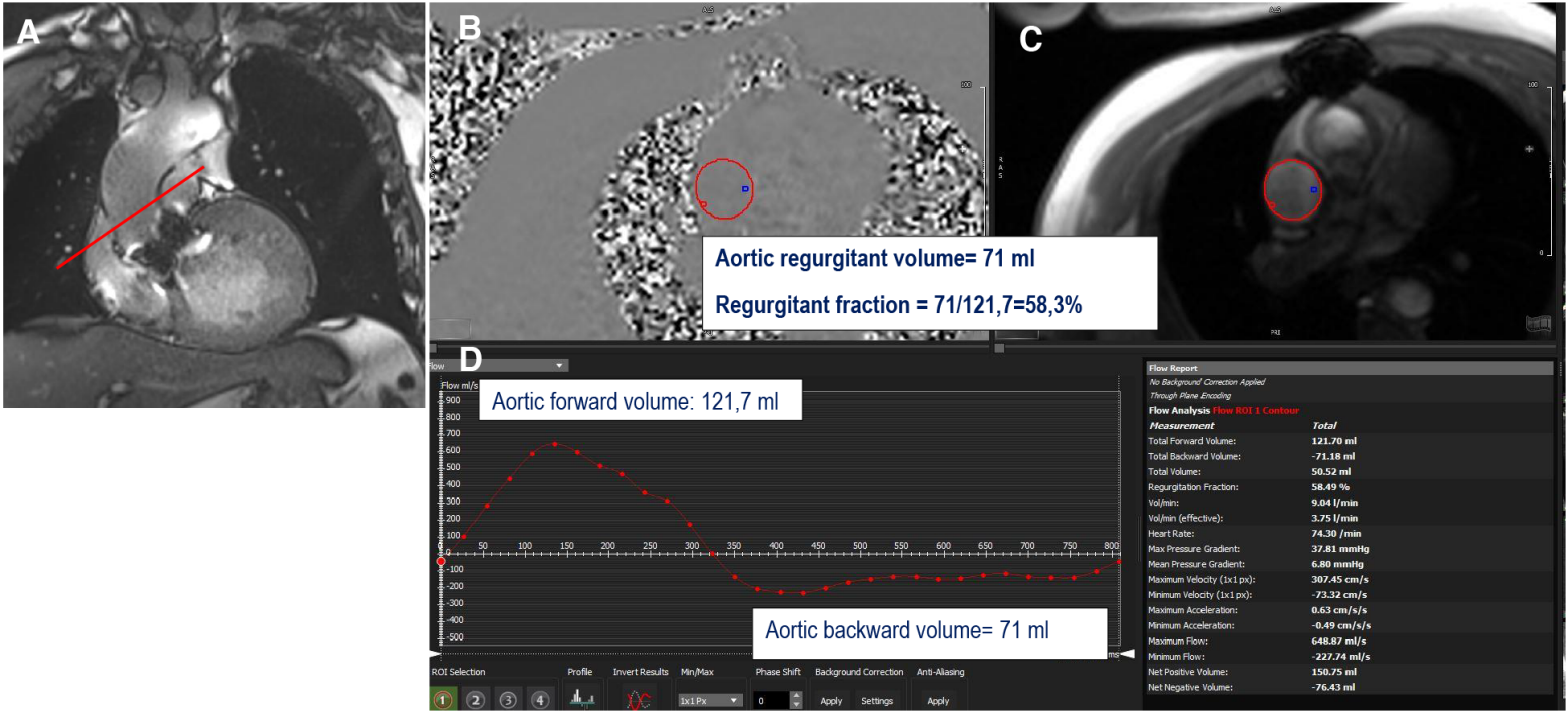
Example of direct quantification of severe aortic regurgitation for a patient with a degenerated surgical aortic prosthesis (perimount). The perpendicular line (red) above the prosthesis (**A**) indicates the scanning position for phase-contrast velocity mapping, generating phase (**B**) and magnitude (**C**) images and a flow curve image (**D**). The aortic regurgitant volume is represented by the area under the diastolic flow curve (71 ml); the regurgitant fraction is calculated as the aortic backward volume/aortic forward volume = 71/121.7 = 58.3%.

Paravalvular aortic leakage (PVL), defined as significant regurgitation between the sealing area of the valve and the adjacent tissue of the annular area of the patient, is a serious post-procedural complication, with a higher incidence in TAVI than SAVR (21, 22). It generally results in TAVI from mispositioning due to calcifications or an undersized valve (23). Following TAVI, PVL is associated with increased mortality (24, 25), and even mild PVL is associated with a poorer prognosis (26). Echocardiography is the first-line modality to assess and quantify PVL. However, its accuracy for the quantification of aortic regurgitation can be limited by eccentric multiple jets and an irregular orifice (27) and tends to underestimate the severity of the regurgitation. CMR is not able to differentiate between intra or paravalvular regurgitation due to the presence of hardware artefacts, but is able to assess and accurately quantify aortic regurgitation, irrespective of jet morphology (28). By CMR, aortic regurgitation appears as a dark jet into the LVOT in the coronal LVOT view or three-chamber view (Supplementary Video S1) and can be quantified using a direct method requiring PC sequences with a plane located in the ascending aorta (above artefacts generated by the aortic prosthesis) perpendicular to the direction of blood flow (29). This sequence generates a magnitude image that allows delineation of the aorta and a phase map encoding the velocities within each voxel. A flow curve is generated allowing calculation of the aortic regurgitant volume (ARvol = area under the backward flow curve during diastole) and regurgitant fraction (RF = ARvol/ aortic forward flow; AFF = area under the forward flow curve) (Figure 6) (Table 1). This method is highly reproducible and valid in cases of coexisting valvular regurgitation but requires correct maximal velocity encoding and careful placement. Two indirect methods can serve for internal validation but are inaccurate in situations of other concomitant valve diseases (9). One is a simple approach using only bSSFP sequences in the short axis and the result is expressed as the difference between the left and right ventricular stroke volume but is prone to errors in right ventricular contouring. The other method requires PC velocity mapping of the main pulmonary artery to measure pulmonary forward flow (PFF). ARvol is expressed as AFF-PFF. In native aortic regurgitation, ARvol > 40 ml and RF ≥ 30% by CMR define moderate to severe regurgitation (9, 30, 31), with a good correlation with echocardiography (32, 33). Recently, Malafji et al. found an optimal threshold for the association of the severity of aortic regurgitation with the outcome of an ARvol of 47 ml and a RF of 43% by CMR (31). After surgical replacement, a recent study using transoesophageal echocardiography and CMR (RF cutoff of 40%) on 31 patients with PVL (23 aortic valve and 8 mitral valve) found a moderate correlation between semiquantitative transoesophageal echography and CMR (*r* = 0.55, *p* < 0.01) (34). These results were independent of the position (aortic or mitral) and type of prosthesis (mechanical or biological), with half of the patients with mild to moderate prosthetic regurgitation reclassified at least one grade or higher by CMR. The underestimation of severity of prosthetic regurgitation by transthoracic or transoesophageal echocardiography was confirmed in patients with TAVI (22, 35). Ribeiro et al. found a poor correlation between CMR and multiparametric transthoracic echography for asymptomatic patients with an expandable prosthetic balloon (Edwards-Sapien®) (*r* = 0.59, *p* < 0.001), with an underestimation of the severity of aortic regurgitation by transthoracic echography in 61.9% of cases using a RF cut off ≥30% by CMR (36). These results are consistent with those of previous studies (37), even when using a higher CMR RF cut off (40%) for symptomatic patients (38), and were confirmed in a recent meta-analysis that pooled the results of seven studies comparing 2D transthoracic echography to CMR (39). The well-documented better accuracy of CMR in detecting and quantifying periprosthetic regurgitation is related to the difficulty of echocardiography to clearly image the regurgitation jet due to acoustic shadowing from the valve stent, as well as the paravalvular nature of the regurgitation, with multiple, eccentric, and irregularly shaped jets. Indeed, quantitative echo indices, such as the diameter of the vena diameter or effective regurgitant orifice area using the convergence method, primarily used to assess native aortic regurgitation (more uniform and central), are clearly less accurate in PVL. By contrast, CMR offers direct and reliable quantification (even in cases with multiple jets), with lower inter- and intra-observer variability (28).

Four-dimensional flow, with its ability to measure blood flow velocities in any orientation in space, could theoretically be an accurate tool to quantify PVL. However, 4D flow imaging requires specific hardware and software as well as an additional training for clinicians to accurately interpret. Additionally, cardiac motion artefacts and the presence of turbulent flow can further complicate the interpretation of 4D flow data. To the best of our knowledge, no data are yet available using this method in patients with biological aortic valves.

Beyond the accuracy and reproducibility of CMR to quantify PVL after the surgical placement of an aortic biological valve, it may also have prognostic value. Ribeiro et al. showed that a RF ≥ 30% by CMR best predicted two-year all-cause mortality and the combined end point of mortality and hospitalization for heart failure in a cohort of 135 patients with residual AR after TAVI followed for a median of 26 months (40). Furthermore, CMR AR grading performed 40 days after the TAVI procedure was associated with a significant additive predictive value of clinical events compared to early (median 6 days) echocardiography (*p* < 0.01).

Although echocardiography is recommended for initial evaluation, CMR appears to be a complementary and a more accurate tool to quantify AR after biological aortic valve replacement and could be implemented for patients with at least moderate or higher PVL or inconclusive transoesophageal or transthoracic echography for any reason (poor echo windows, difficult AR quantification).

### Cine short-axis view for left ventricular reverse remodelling

Chronic aortic valve dysfunction generates volume and pressure overload, leading to concentric or eccentric LV remodelling due to an increase in LV wall thickness, LV mass, myocyte size, and extracellular matrix expansion (41). After aortic valve replacement or TAVI, LV mass and hypertrophy decrease by 20% to 30% within a year after the procedure due to the decrease in ventricular afterload (42–44). The regression of hypertrophy and ventricular volume, also called reverse remodelling, is associated with better survival than a lack of improvement (45–47). These cardiac structural changes are generally evaluated by echocardiography. LV mass quantification uses geometric assumptions validated in the normal heart but has a tendency to overestimate LV mass in the presence of asymmetric LV hypertrophy (48, 49). CMR, is the gold standard method to measure LV mass and wall thickness due to its ability to measure ventricular volume and mass without the need for geometric assumptions and is more accurate and reproducible than echocardiography (49). bSSFP images are acquired on a stack of short-axis views that include both ventricles from the base to the apex, allowing endocardial and epicardial contouring at the end-diastole and end-systole.

A recent meta-analysis that included 305 patients who completed pre- and post-TAVI CMR showed a significant reduction in left ventricular end diastolic volume (median reduction of 4 ml/m^2^), left ventricular end systolic volume (median reduction of 5 ml/m^2^), left ventricular mass index (median reduction of 15.1 g/m^2^), and increased LV ejection fraction (median increase of 3.4%) within six months after TAVI (50). Such reverse remodelling is similar for SAVR and TAVI (the first and second generation) (51).

### T1 mapping and late gadolinium enhancement for myocardial structure changes after procedure

Unlike echocardiography, CMR has the unique and remarkable ability to assess focal interstitial fibrosis [using late gadolinium enhancement (LGE)] and diffuse interstitial fibrosis [using T1 mapping and measurement of the extracellular volume fraction (ECV)] for a better understanding the regression of left ventricular hypertrophy (LVH) after the correction of pressure overload due to AS. LVH combines cell and matrix compartments (Table 1). The ECV represents the volume of the heart not occupied by cells and the intracellular volume (ICV = 1-ECV) reflects the cell volume fraction, which reveals cell hypertrophy. Treibel et al. showed a greater reduction in the cell volume (22%) than matrix volume (ECV x LV mass index) (16%) for 116 patients one year following SAVR (52). A smaller study with a pure model of interstitial diffuse fibrosis (without focal fibrosis) showed that a reduction in cellular hypertrophy is the earliest change, occurring from six months after SAVR, followed by a reduction in the volume of the interstitial matrix (53).

Focal fibrosis, represented by focal LGE by CMR is believed to be irreversible (52, 54) and is associated with cardiovascular mortality for both sexes, even after SAVR (55). These observations have raised the hypothesis that early valve intervention for patients with asymptomatic severe AS and mid-wall fibrosis could improve their prognosis. Results from The Evolved-AS study (early valve replacement guided by biomarkers of LV decompensation in asymptomatic patients with severe AS) should address this important issue.

### Longitudinal strain

Baseline global longitudinal strain assessed by echocardiography has been shown to be a strong predictor of a decrease in LVH after TAVI for patients with severe AS (56). From standard cine sequences, CMR is able to assess global and regional strain in the same way as echocardiography. Reduced longitudinal strain by CMR has been found for patients with AS relative to normal controls and improved after TAVI (57), except for patients with paradoxical low flow/low gradient AS (58).

### CMR is not the best tool to assess thrombi and pannus formation

Although the risk of valve thrombosis or pannus formation is low (59), it is crucial to differentiate these two complications for appropriate and rapid treatment (anticoagulation, surgery, or thrombolysis). Echocardiography and CCT are the preferred imaging modalities to diagnose these complications due to the lower spatial resolution of CMR, the inability to detect calcifications, and the presence of artefacts related to the metal component of the prosthesis. Thus far, there have been no studies to evaluate the usefulness of CMR in assessing thrombi or pannus.

## Conclusion

Transthoracic and transoesophageal echocardiography have an indisputable role in following patients with biological aortic valves and in detecting deterioration. CMR represents a complementary tool when the results using these modalities are equivocal or inconclusive for any reason.

The added value of CMR is predominantly in assessing aortic regurgitation (paravalvular and intravalvular, particularly for patients with TAVI) and in evaluating the impact of valve intervention on left ventricular myocardial structural changes. Using a direct method, aortic regurgitation can be quantified with higher accuracy than by echocardiography and should be performed for patients with moderate or higher regurgitation, especially if an additional intervention is discussed. Using cine images and LV reverse remodelling, a prognostic factor after aortic valve intervention can be accurately assessed. Using LGE sequences and T1 mapping, CMR plays a role in assessing focal and interstitial diffuse fibrosis before and after aortic procedures for a better understanding of the regression of LVH and the patient's prognostic stratification.

## Supplementary material

Supplementary Video 1 Cine three-chamber view showing aortic regurgitation (dark jet into LVOT) for a patient with a degenerated surgical stented aortic prosthesis (Mosaic).
